# Case Report: Clinical and genetic analysis of a family with hereditary spherocytosis combined with familial chylomicronemia syndrome

**DOI:** 10.3389/fgene.2026.1659838

**Published:** 2026-01-23

**Authors:** Yumei Qin, Yanping Liu, Kecheng Li, Yaoming Deng, Hualian Li, Xiao Chen, Xuan Pan, Xiaojing Huang, Mengyue Xie, Xingjiang Long, Shifu Tang

**Affiliations:** 1 Department of Laboratory Medicine, Key Laboratory of Precision Medicine for Viral Diseases, Guangxi Health Commission Key Laboratory of Clinical Biotechnology, Liuzhou People’s Hospital, Liuzhou, China; 2 Blood Transfusion Department, Liuzhou People’s Hospital, Liuzhou, China; 3 Department of Pediatrics, Liuzhou People’s Hospital, Liuzhou, China

**Keywords:** familial chylomicronemia syndrome, hereditary spherocytosis, LPL gene, novel mutation, SPTB gene

## Abstract

**Objective:**

This study was conducted to investigate the clinical and genetic characteristics of a family affected by hereditary spherocytosis (HS) combined with familial chylomicronemia syndrome (FCS), identify the pathogenic cause, and provide a basis for the clinical diagnosis, treatment, and genetic counseling of affected children.

**Methods:**

Clinical data were collected from family members. High-throughput sequencing was performed to identify pathogenic variants in genes associated with HS and FCS in the proband. Suspected pathogenic mutations were confirmed in family members via PCR-Sanger sequencing. Bioinformatics analysis and three-dimensional protein structure prediction were also conducted.

**Results:**

The proband presented with severe anemia, splenomegaly, and jaundice. Genetic testing revealed a heterozygous mutation, c.6005G>A (p.Trp2002*), in the spectrin beta chain (*SPTB*)gene (NM_001355436.2) and a missense mutation, c.292G>A (p.Ala98Thr), in the lipoprotein lipase (*LPL*) gene (NM_000237.3). The *SPTB* c.6005G>A (p.Trp2002*) mutation was inherited from the mother, who exhibited mild anemia, jaundice, and splenomegaly. The *LPL* c.292G>A (p.Ala98Thr) mutation was inherited from the father, who had hypertriglyceridemia. The *SPTB* c.6005G>A (p.Trp2002*) mutation is extremely rare in the general population.

**Conclusion:**

The heterozygous mutations *SPTB* c.6005G>A (p.Trp2002*) and *LPL* c.292G>A (p.Ala98Thr) are the pathogenic causes in this family and provide a basis for clinical management and genetic counseling. Based on the HGMD, 1000G, and ExAC databases, the *SPTB* c.6005G>A (p.Trp2002*) mutation is reported here for the first time, enriching the mutation spectrum of the *SPTB* gene.

## 1 Introduction

Hereditary spherocytosis (HS) is a globally reported disease and the most common type of genetic anemia among people of Nordic descent, with a prevalence rate of about 1/2000 in the Nordic population (Behling-Kelly et al., 2014). There are significant geographical differences in prevalence rates, which are relatively rare in Asian populations. It has been reported that the crude incidence of HS in Korea was 1 in every 5000 births (Behling-Kelly et al., 2014). Recently, China has reported cases of HS, with an estimated prevalence rate of 1:100,000 (Sun et al., 2019). It is important to note that this figure may be an underestimation, as mild cases are often undiagnosed or under-diagnosed in clinical practice. HS is among the most common genetically inherited hemolytic disorders, resulting from mutations in genes encoding red blood cell membrane proteins. Approximately 75% of cases exhibit autosomal dominant inheritance, whereas the remainder may follow an autosomal recessive pattern (Bolton-Maggs et al., 2012; Tole et al., 2020). Clinical manifestations include anemia, jaundice, and splenomegaly (Tole et al., 2020). Five pathogenic genes associated with HS have been identified, including *SPTA1*, *ANK1*, *SPTB*, *SLC4A1*, and *EPB42*, encoding α-spectrin, ankyrin, β-spectrin, band 3 protein, and protein 4.2, respectively (Polizzi et al., 2025; Perrotta et al., 2008). These gene mutations lead to abnormal membrane anchoring on the surface of red blood cells, altering their normal biconcave disc shape and resulting in spherocytosis (Sun et al., 2019). Spherocytes have poor deformability and are easily destroyed when they pass through the splenic sinus, leading to hemolytic anemia.

Familial chylomicronemia syndrome (FCS, OMIM 238600), also known as type 1 hyperlipoproteinemia, is a rare autosomal recessive disorder (Stroes et al., 2024). In 2018, Khavandi et al. (2018) analyzed 385,000 electronic medical records from 2008 to 2017 in New York State and found that the incidence rate of FCS was approximately 1/100,000. Pallazola et al. (2020) retrospectively analyzed 1,627,763 patients who visited Johns Hopkins Hospital from 2013 to 2017 and calculated a prevalence rate of FCS as high as 13/1 million. Shah et al. (2018) retrospectively analyzed 70,201 patients who visited the Lipid Center at the Cleveland Clinic from 2006 to 2016. They found that the prevalence rate of FCS was at least 1/5,000, which was 200 times higher than the reported incidence rate. Currently, there are no relevant data reports on the incidence rate of FCS in China. It results mainly from defects in the lipoprotein lipase (*LPL*) and its cofactor apolipoprotein C-II (ApoC2), which are responsible for releasing free fatty acids from triglycerides (TG) in dietary-derived chylomicrons and hepatic very low-density lipoprotein (VLDL), allowing free fatty acids to be internalized in the heart or skeletal muscle for energy production or in adipose tissue for energy storage. Therefore, defects in these two proteins result in impaired metabolism of triglyceride-rich chylomicrons (Wojtukiewicz et al., 1999). Characteristic features include severe hypertriglyceridemia (HTG) (TG > 11.3 mmol/L), recurrent episodes of acute pancreatitis, cutaneous xanthomas, and hepatosplenomegaly (Ba et al., 2020; Brinton et al., 2025). Family analyses and genome-wide association studies have linked type 1 hyperlipoproteinemia to mutations in *LPL*, *APOC2*, *APOA5*, *LMF1*, *GPIHBP1*, and *SARA2*, with *LPL* mutations being the most common (Gallo et al., 2020). No clinical reports have mentioned patients concurrently affected by HS and FCS, which may lead to misdiagnosis or missed diagnosis. In this study, we analyzed the clinical and genetic characteristics of a family affected by HS and FCS, providing references for clinical practice and genetic counseling.

## 2 Materials and methods

### 2.1 Clinical data

**FIGURE 1:**
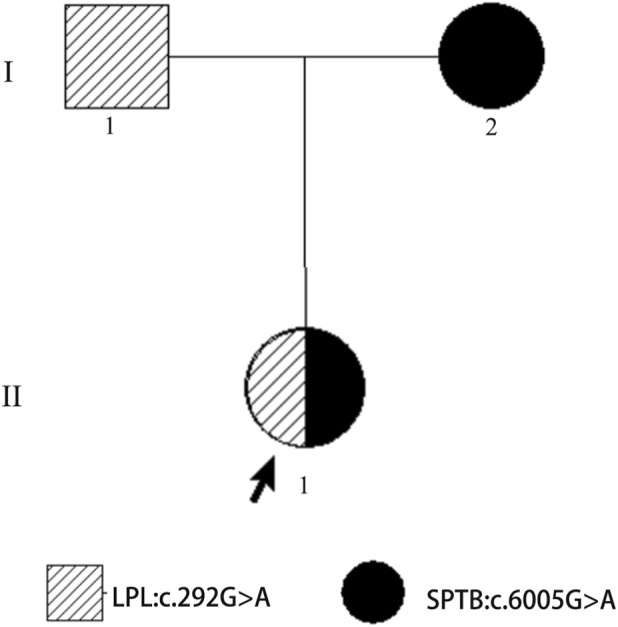
Pedigree of a Chinese family with hereditary spherocytosis and type1 hyperlipoproteinemia. Black arrow: proband (Ⅱ-1); square: male (I-1,proband’s father); circle: female (I-2,proband’s mother).

The proband (Figure 1, II-1), a female born in October 2011, was admitted due to “pallor for more than 7 years.” Physical examination revealed an anemic appearance, no prominent signs of jaundice of the skin or sclera, no xanthomas, no lymphadenopathy, normal breath sounds in both lungs, a liver not palpable below the costal margin, and a spleen palpable 3.0 cm below the ribs. The parents were non-consanguineous, and there was no family history of obesity, diabetes, hypertension, or cardiovascular disease. The mother presented with mild anemia, jaundice, and splenomegaly. The father’s plasma appeared milky white.

### 2.2 Sample collection

This study was approved by the Ethics Committee of Liuzhou People’s Hospital Affiliated with Guangxi Medical University (NO: KY 2024-004-01). After informed consent was obtained from the proband’s parents, 2 mL peripheral venous blood samples were collected from the proband and her parents using EDTA anticoagulant tubes. Genomic DNA was extracted from peripheral blood using a DNA extraction kit (Beijing Tiangen Biotech (Beijing) Co., Ltd.).

### 2.3 Whole-exome sequencing

Genomic DNA from the proband and her parents was sent to Guangzhou KingMed Diagnostics Laboratory for genetic testing. Captured DNA samples were used to perform whole-exome sequencing using the Illumina HiSeq2500 platform to detect sequence variations in all exonic and splice site regions of genes associated with HS and FCS. The sequencing data were analyzed using the GATK software suite. The reads were aligned to the UCSC hg19 reference genome using BWA. Variant annotation was performed via VEP software. Variants were filtered, and their pathogenicity was predicted and classified based on databases, including ClinVar, OMIM, and HGMD, as well as population databases such as gnomAD.

### 2.4 PCR-Sanger sequencing validation

Two suspected mutation sites identified by high-throughput sequencing were confirmed in the proband and her parents via PCR-Sanger sequencing. The primers were designed using Premier 5.0 software. For the *SPTB* gene, the primers used were as follows: forward primer, 5′-GCG​TGT​CTA​CTC​TGG​CCT​TCT-3′; reverse primer, 5′-GAG​ATC​GGG​GCT​ACA​CAC​AG-3′. For the *LPL* gene, the forward primer was 5′-TTT​TTC​CAT​TTC​ATG​CAG​GTG-3′, and the reverse primer was 5′-CCC​AGT​CTT​ACC​TCC​ATC​CAG-3′. PCR was performed using DNA from the proband and her parents in a 50 μL reaction containing 25 μL of Taq polymerase master mix, 1 μL each of forward and reverse primers, 2 μL of template DNA, and nuclease-free water to a final volume. The PCR cycling conditions were as follows: initial denaturation at 95 °C for 4 min; 35 cycles of 95 °C for 30 s, annealing for 30 s, and extension at 72 °C for 30 s; and final extension at 72 °C for 8 min. PCR products were stored at 4 °C, purified, and sequenced.

### 2.5 Pathogenicity prediction and conservation analysis of gene mutations

Novel mutation sites were assessed for pathogenicity via Mutation Taster, PolyPhen-2, PROVEAN, SIFT, and NMDetective software. The conservation of amino acid sequences around mutation sites was analyzed across species using the DNAMAN software.

### 2.6 Protein structure prediction

Homology modeling of the *LPL* gene c.292G>A (p.Ala98Thr) mutation was performed using the PyMOL software to predict the effect of changes in amino acid residues on protein function.

## 3 Results

### 3.1 Laboratory test results of the proband and her parents

**FIGURE 2:**
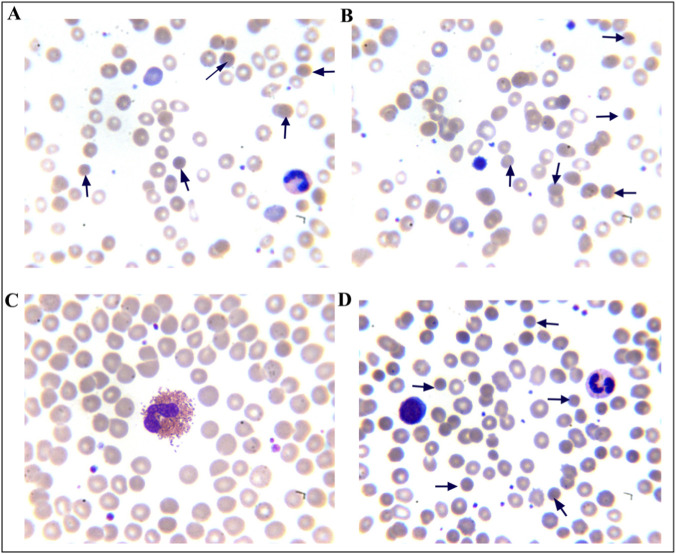
Morphology of peripheral blood erythrocytes (Wright–Giemsa, magnification, ×1000). The arrow shows a typical spherical erythrocytes. **(A,B)** Proband; **(C)** Father; **(D)** Mother.

The laboratory test results of the proband and her parents revealed that the proband and her mother had lower levels of Hb, MRV, and MSCV. In contrast, RET, IBIL and osmotic fragility of red blood cells levels were higher. The proband had mildly elevated TG levels, whereas her father’s TG level was considerably high at 19.2 mmol/L (Table 1). A peripheral blood smear revealed 9.5% spherocytes in the proband and 20.5% in her mother, whereas no abnormalities were observed in the father’s peripheral blood smear (Figure 2). Based on clinical features, laboratory tests, and genetic findings, the proband was diagnosed with HS combined with FCS. Three months later, the proband underwent splenectomy. At the 1-month postoperative follow-up, her Hb level had increased to 106.00 g/L, and her TG level was 5.4 mmol/L.

**TABLE 1:** Laboratory findings of the proband and his family members.

Characteristic	Proband	Father	Mother	Reference
RBC(×1012/L)	2.02	5.45	3.74	3.50–5.50
Hb (g/L)	51.80	169.40	109.50	110.00–160.00
MCH (pg)	25.61	31.28	29.31	27.00–34.00
MCHC (g/L)	303.90	336.10	339.40	316.00–354.00
MCV (f l)	84.27	93.05	86.35	82.00–100.00
MSCV (f l)	52.30	75.50	54.50	84.00–104.00
MRV (f l)	80.60	101.60	76.50	101.00–119.00
TBIL (μmol/L)	84.8	8.2	47.3	3.40–20.50
IBIL (μmol/L)	75.4	6.7	28.6	3.10–14.30
RET (%)	14.3	1.4	15.6	0.5–1.5
T C (mmol/L)	4.6	5.71	2.98	2.8–5.18
T G (mmol/L)	5.2	19.2	0.83	0.6–1.7
HDL-C (mmol/L)	1.27	0.68	1.33	1.04–1.55
LDL-C (mmol/L)	2.98	1.72	3.21	1.56–3.37
Thalassemia gene testing	Negative	Negative	Negative	Negative
Activity of G-6-PD (U/L)	4234	4215	2893	>1300
Direct antiglobulin test	Negative	Negative	Negative	Negative
Haemolysis begins (g/L)	5.6	3.8	5.2	3.80–4.60
Haemolysis complete (g/L)	3.2	2.8	2.8	2.80–3.20

RBC, red blood cell; Hb, hemoglobin; MCH, mean corpuscular hemoglobin; MCHC, mean corpuscular hemoglobin concentration; MCV, mean corpuscular volume; MSCV, mean sphered corpuscular volume; MRV, mean reticulocyte volume; TBIL, total bilirubin; IBIL, indirect bilirubin;RET, reticulocyte; TC, total cholesterol; TG, triglyceride;HDL-C, high density lipoprotein cholesterol; LDL-C, low-density lipoprotein cholesterol.

### 3.2 Genetic testing results

**FIGURE 3:**
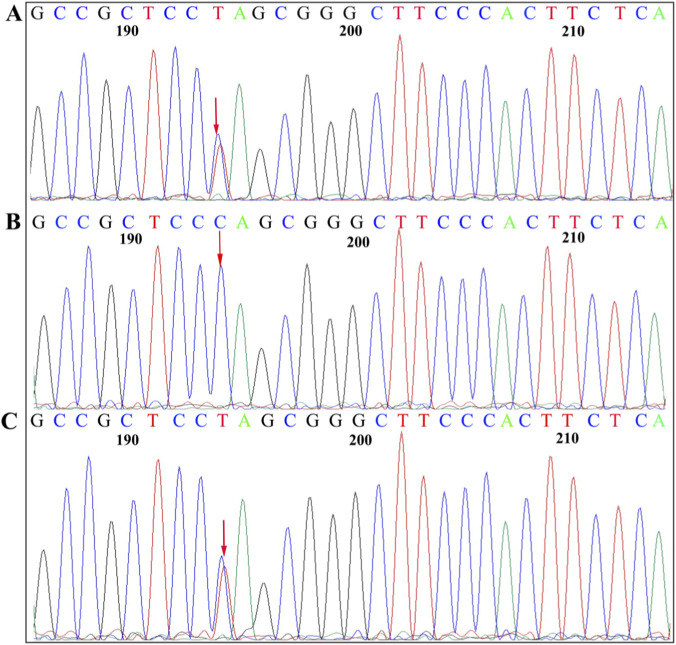
Sanger sequencing of the *SPTB* c.6005G>A (p.Trp2002*) mutation. The sequence shown is that of the anti-sense strand. Arrows, mutation sites. **(A)** Proband, heterozygous mutation. **(B)** Father, normal. **(C)** Mother, heterozygous mutation.

**FIGURE 4:**
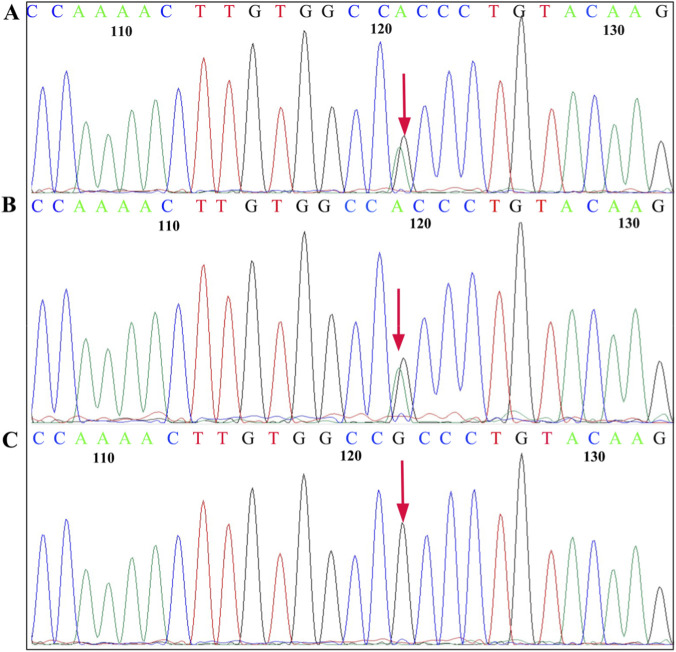
Sanger sequencing of the *LPL* c.292G>A (p. Ala98Thr) mutation. The sequence shown is that of the sense strand. Arrows, mutation sites. **(A)** Proband, heterozygous mutation. **(B)** Father, heterozygous mutation. **(C)** Mother, normal.

Genetic testing revealed that the proband carried a heterozygous mutation c.6005G>A (p.Trp2002*) in the *SPTB* gene (NM_001355436.2) and a missense mutation, c.292G>A (p.Ala98Thr), in the *LPL* gene (NM_000237.3). She inherited the *SPTB* gene c.6005G>A (p.Trp2002*) mutation from her mother (Figure 3) and the *LPL* gene c.292G>A (p.Ala98Thr) mutation from her father (Figure 4).

### 3.3 Pathogenicity prediction and conservation analysis of gene mutations

**FIGURE 5:**
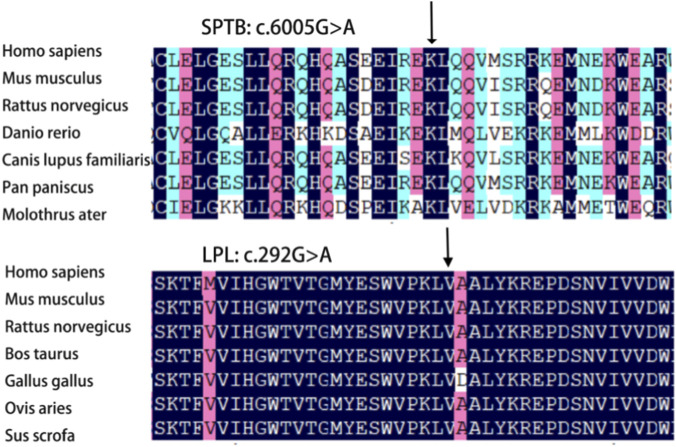
Conservative analysis of *SPTB* gene c.6005G>A (p.Trp2002*) and *LPL* gene c.292G>A (p.Ala98Thr) among different species. The black arrow indicates that the mutation is located at a highly conserved amino acid site.

The *SPTB* gene c.6005G>A (p.Trp2002*) mutation is a nonsense mutation that was not previously reported in the HGMD and represents a novel mutation. The *LPL* gene c.292G>A (p.Ala98Thr) mutation is a missense mutation classified as pathogenic in the ClinVar database. Pathogenicity prediction was performed using online software, and the results are presented in Table 2. Conservation analysis revealed that *SPTB* c.6005G>A (p.Trp2002*) and *LPL* c.292G>A (p.Ala98Thr) mutation sites are highly conserved across species (Figure 5).

**TABLE 2:** Online software predicts pathogenicity of mutation sites.

Gene	Mutation	Protein	Mutation taster	PROVEAN	SIFT	PolyPhen-2
SPTB	c.6005G>A	(p.Trp 2002*)	Disease-causing	Deleterious	NA	NA
*LPL*	c.292G>A	(p.Ala98Thr)	Disease-causing	Deleterious	Damaging	Probably damaging

Abbreviations: NA, not applicable.

### 3.4 Protein structure analysis of the LPL c.292G>A mutation

**FIGURE 6:**
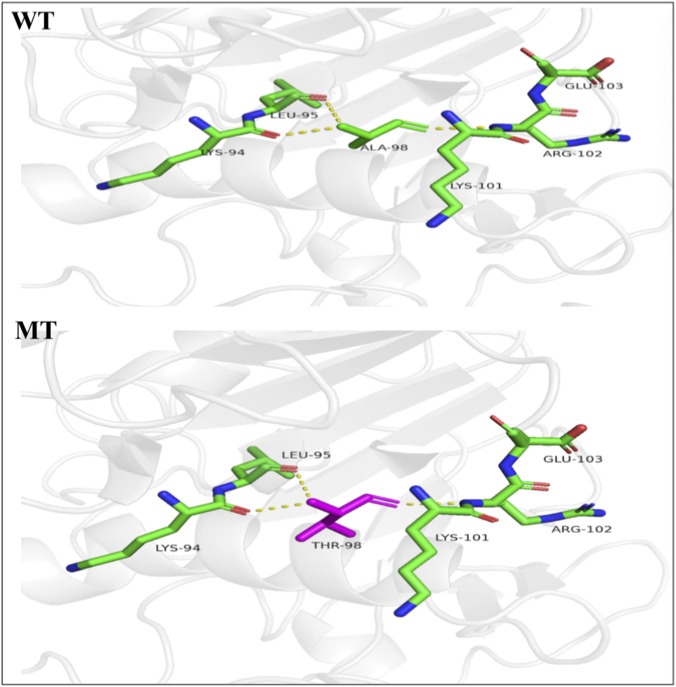
Protein structure analysis of *LPL* c.292G>A (p. Ala98Thr). WT: structure of the wild-type LPL catalytic domain. The alanine residue at position 98 is situated within a hydrophobic core, contributing to stable folding. MT: structure of the mutant LPL (p.Ala98Thr). Substitution with threonine introduces a residue with greater molecular weight, steric hindrance, and hydrogen bonding capacity, while reducing local hydrophobicity. This alteration is predicted to distort the spatial arrangement of key residues, leading to protein destabilization, reduced enzymatic activity, and decreased secretion.

Protein structure analysis of the *LPL* gene c.292G>A (p.Ala98Thr) mutation revealed that, compared with the wild-type residue, the mutated residue has a greater molecular weight, lower hydrophobicity, greater hydrogen bonding ability, and greater steric hindrance. This mutation may alter the spatial arrangement of key residues, reducing enzyme activity and decreasing protein secretion (Figure 6).

## 4 Discussion

Hereditary spherocytosis (HS), a common inherited hemolytic disorder (Turpaev et al., 2025), is associated with the five pathogenic genes: *SPTB, ANK1, SPTA1, SLC4A1,* and *EPB42*. Anemia, jaundice, and splenomegaly are common in patients with moderate to severe HS, whereas milder forms may present with subtle or no symptoms (Wu et al., 2024). The diagnosis of HS can be made according to the previous described criteria (Wu et al., 2021). In this study, the proband presented with severe anemia, splenomegaly, and jaundice; increased spherocytes in the peripheral blood smear; MRV≤95.77 fL; MSCV < MCV; and a higher reticulocyte ratio and indirect bilirubin levels. The proband’s mother had the same clinical manifestations and laboratory results, with a positive family history. High-throughput sequencing revealed a heterozygous *SPTB* c.6005G>A (p.Trp2002*) mutation in the proband, with maternal inheritance confirmed by PCR-Sanger sequencing. The SPTB c.6005G>A (p.Trp2002*) mutation has an extremely low frequency in the general population. It is absent from the gnomAD database (v4.1.0), which includes sequence data from over 140,000 exomes and 15,000 genomes, indicating an estimated global allele frequency of <3.6 × 10^−6^. This variant has also not been reported in the HGMD or 1000G databases, enriching the mutation spectrum of the SPTB gene (Wang et al., 2024; More and Kedar, 2025; Panarach et al., 2024). Known mutation types in *SPTB* include nonsense, frameshift, splice-site, and missense mutations (Panarach et al., 2024). In this study, the *SPTB* c.6005G>A (p.Trp2002*) mutation is located at the C-terminus of β-spectrin Repeat 17, where the 2002nd amino acid tryptophan is changed to a stop codon. Mutation Taster and PROVEAN predicted this mutation as likely pathogenic. The results of amino acid conservation analysis revealed that the mutation site is highly conserved in the β-subunit. Additionally, NMDetective predicted that the mutation may lead to nonsense-mediated mRNA decay, resulting in β-spectrin haploinsufficiency or premature translation termination. This can produce truncated proteins with or without actin-binding, dimerization, ankyrin-binding, and tetramerization domains, thereby impairing the function of spectrin. This functional disruption further compromises the critical role of β-spectrin, leading to decreased stability of the erythrocyte membrane skeleton. The loss of membrane stability promotes the transformation of red blood cells into spherocytes, which are subsequently cleared prematurely in the spleen and peripheral blood. This pathogenic cascade ultimately explains the patient’s clinical manifestations of hemolytic anemia, splenomegaly, and the presence of increased spherocytes in the peripheral blood smear.

The clinical manifestations of FCS include severe HTG, recurrent abdominal pain, acute pancreatitis, eruptive xanthomas, hepatosplenomegaly, and chylous plasma, among others (Stroes et al., 2024; Chyzhyk and Brown, 2020). Pathogenic genes associated with FCS include *LPL, APOC2, GPIHBP1, LMF1*, and *APOA5,* among which mutations in the *LPL* gene account for more than 90% of cases (Brinton et al., 2025; Gallo et al., 2020; Ariza and Valdivielso, 2022). In this study, the proband had slightly higher TG, normal total cholethe diagnosissterol (TC), and splenomegaly, with inconspicuous clinical manifestations, that can be missed. The proband’s father had milky plasma with TG levels as high as 18.2 mmol/L and reduced HDL-C. He did not have obesity, diabetes, hypertension, or a family history of cardiovascular diseases. A rare monogenic cause of FCS was suspected, and subsequent *LPL* gene analysis confirmed the clinical suspicion. The proband carried the *LPL* gene c.292G>A (p.Ala98Thr) mutation inherited from the father. The proband’s TG was slightly elevated, which confirmed that carriers of pathogenic *LPL* mutations may present with mild HTG (Bolton-Maggs et al., 2012). The c.292G>A (p.Ala98Thr) variant carried by the proband is a missense mutation in the coding region of the *LPL* gene. The LPL c.292G>A (p.Ala98Thr) variant is rare, as evidenced by its low allele frequency of 4.40 × 10^−5^ in the gnomAD database (v4.1.0), derived from 71 observed alleles among 1,614,010 sequenced. The absence of homozygous individuals further supports its rarity. Therefore, it is considered a rare variant that may underlie the patient’s condition. This variant has been reported in multiple patients with hyperlipidemia, and functional studies have shown that this mutation reduces the catalytic and secretory activity of *LPL* (Chen et al., 2014; Xi et al., 2015; Chan et al., 2002). On the other hand, hyperlipidemia is known to increase RBC fragility. In animal models, erythrocyte osmotic fragility has been reported in dogs with hyperlipidemia and dyslipidemia (Behling-Kelly et al., 2014). As for the underlying mechanism by which hyperlipidemia affect erythrocyte osmotic fragility, it has been proposed that hyperlipidemia may generate reactive oxygen species and other free radicals, thereby increasing the autoxidation rate of Hb and promoting the partial conversion of HbO2 and unstable Hb molecules into Met-Hb and carboxyhemoglobin. The increase in erythrocyte osmotic fragility may be attributed to the interference with ion movement through the membrane and changes in the molecular properties of membrane macromolecules (Abdelhalim and Moussa, 2010). In this study, the proband had mildly elevated TG levels, whereas her father’s TG level was considerably high. Notably, neither individual was on any lipid-lowering medication, indicating that the observed hypertriglyceridemia directly reflects the genetic defect without confounding pharmacological influence. A peripheral blood smear revealed 9.5% spherocytes in the proband, and the red blood cell osmotic fragility test is increased, which is consistent with the morphological changes in HS patients. Interestingly, despite the father’s more severe hypertriglyceridemia, his peripheral blood smear and osmotic fragility test were normal. This discrepancy suggests that the relationship between hyperlipidemia and RBC fragility may not be straightforward or dose-dependent in humans and could be modulated by additional factors. Therefore, whether the increased osmotic fragility in the proband is attributable solely to dyslipidemia or primarily to her concurrent hereditary spherocytosis requires further exploration.

The *LPL* gene is located on chromosome 8p22. It spans 30 kb and contains 10 exons and nine introns. It encodes a protein containing 475 amino acids (Huang et al., 2022; Cao et al., 2018). Mutations in the *LPL* gene can result in varying degrees of reduced *LPL* enzyme activity, thereby impairing the metabolism of TG-rich chylomicrons. This condition manifests as turbid serum or plasma and severely elevated fasting TG levels (Gilbert et al., 2001). Mutation types in *LPL* include nonsense, frameshift, splice-site, and missense mutations, among which missense mutations are the most common, accounting for about 75% (Ariza Corbo et al., 2024). In this study, the proband carried the *LPL* gene c.292G>A (p.Ala98Thr) missense mutation, resulting in an amino acid change from alanine to threonine at position 98. Polyphen-2, mutation tester, and SIFT software predictions suggested that this mutation site is probably pathogenic, and conservation analysis revealed that the site is highly conserved across species. Additionally, protein structural analysis indicated that, compared to the wild-type residue, the *LPL* gene c.292G>A (p.Ala98Thr) mutation results in a mutant residue with greater molecular weight, reduced hydrophobicity, increased hydrogen bonding capacity, and greater steric hindrance. This mutation may alter the spatial arrangement of key residues, reducing enzyme activity and protein secretion.

The proband harbored heterozygous mutations in *SPTB* c.6005G>A and *LPL* c.292G>A, which were inherited from the mother and father, respectively, and was diagnosed with HS combined with FCS. As a carrier of the *LPL* c.292G>A (p.Ala98Thr) mutation, the proband presented only mildly elevated TG levels compared with the father’s typical severe chylomicronemia. This milder phenotype may be due to lower dietary fat intake and sex hormone levels, which mitigate lipid accumulation caused by *LPL* deficiency, or to hypersplenism, which accelerates the clearance of ApoB-containing lipoproteins. The proband’s HS clinical symptoms were more severe than those of the mother, probably because the *SPTB* c.6005G>A and *LPL* c.292G>A mutations synergistically exacerbate the proband’s HS symptoms through mechanisms including membrane lipid disorders, oxidative stress, and deterioration of the splenic microenvironment, which requires further functional validation. The proband underwent splenectomy, and at the 1-month post-operative follow-up, the hemoglobin level increased to 106.00 g/L, and the TG level decreased to 5.4 mmol/L. The clinical recommendations for the father included a low-fat diet and regular lipid monitoring.

## 5 Conclusion

To summarize, the heterozygous mutations *SPTB* c.6005G>A (p.Trp 2002*) and *LPL* c.292G>A (p.Ala98Thr) were the pathogenic causes in this family. The proband was diagnosed with HS combined with FCS, providing a basis for clinical diagnosis, treatment, and genetic counseling. HS and FCS are two distinct genetic diseases, characterized by abnormalities in the red blood cell membrane and lipid metabolism disorders, respectively. No direct correlation was found between them. However, hyperlipidemia is known to increase RBC fragility, which might also contribute to anemia (Behling-Kelly et al., 2014).

In this study, we identified a family bearing these two congenital conditions. However, we were not able to find any connections between these two conditions. Whether this is a coincidence or due to currently unknown factors remains to be further investigated.

## Data Availability

The datasets presented in this article are not readily available because of ethical and privacy restrictions. Requests to access the datasets should be directed to the corresponding authors.
